# Efficacy and safety of bevacizumab in neoadjuvant and concurrent chemoradiotherapy for refractory cervical cancer patients

**DOI:** 10.17305/bb.2024.10528

**Published:** 2024-12-01

**Authors:** Hua Yang, Shi Gao Huang, Mohan Dong, Xiaomeng Wang, JunHua He, Huyan Su, Changhao Liu, Yong Zhu, Lichun Wei, Zi Liu

**Affiliations:** 1 Department of Radiation Oncology, The First Affiliated Hospital of Xi’an Jiaotong University, Xi’an, China; 2 Department of Radiation Oncology, The First Affiliated Hospital of Air Force Medical University, Xi’an, China; 3 Department of Medical Education, The First Affiliated Hospital of Air Force Medical University, Xi’an, China; 4 Department of Radiation Oncology, 986 Hospital of Air Force Medical University, Xi’an, China; 5 Tumor Diagnosis and Treatment Center, The First Hospital of Yulin, Yulin, China; 6 Department of Radiotherapy, Baoji Central Hospital, Baoji, China

**Keywords:** Refractory cervical cancer (CC), bevacizumab, neoadjuvant chemotherapy (NACT)

## Abstract

A platinum-based concurrent chemoradiotherapy (CCRT) is the standard treatment for refractory cervical cancer (CC). However, the recurrence of disease and the occurrence of metastasis remain prevalent. We observed the long-term efficacy and safety of bevacizumab combined with neoadjuvant chemotherapy (NACT) and CCRT in refractory CC. A total of 62 patients with refractory CC were enrolled in this study from January 2016 to December 2019. The NACT regimen included bevacizumab (7.5 mg/kg), docetaxel (75 mg/m^2^), and cisplatin (75 mg/m^2^), administered tri-weekly for two cycles. The CCRT regimen included bevacizumab (7.5 mg/kg) and cisplatin (75 mg/m^2^), administered tri-weekly for two cycles. A dose of 45–50 Gy was prescribed for external beam radiotherapy (EBRT), while 30–35 Gy in 4–5 fractions was prescribed for brachytherapy (BT). Among the patients, 21 patients (33.9%) were at stages IIB-IIIB, 8 patients (12.9%) were at stage IIIC1, 19 patients (30.6%) were at stage IIIC2, and 14 patients (22.6%) were at stage IVB. Pelvic, para-aortic, supraclavicular, and inguinal lymph node metastases were discovered in 41 patients (66.1%). The median follow-up was 49.8 months (12.3–82.7 months). The median tumor volumes pre-treatment, after NACT, and before BT were 84.64 ± 53.15 cm^3^, 1.64 ± 13.15 cm^3^, and 0 ± 1.5 cm^3^, respectively. Complete clinical response (cCR) rates after NACT and EBRT were 35.5% and 66.1%, respectively. Four years after the diagnosis, the overall survival (OS) rate was 78.6%, the local region-free survival (LRFS) rate was 91.3%, the disease-free survival (DFS) rate was 70.6%, and the distant metastasis-free survival (DMFS) rate was 81.4%. A total of 29 patients (46.8%) experienced grade 3/4 hematological toxicity, three patients (4.8%) experienced grade 3 gastrointestinal toxicities, and none experienced grade 5 adverse events. Bevacizumab combined with NACT and CCRT significantly improved cCR and OS in refractory CC with acceptable toxicity.

## Introduction

Cervical cancer (CC) is the fourth most common female malignancy worldwide, and its incidence varies widely between countries [1]. A national cervical screening program was introduced in 1988 by the National Health Service (NHS) England in order to combat the burden of the disease. Since then, over a third of cases have been reduced in England [2]. However, there were still 113,400 new CC cases and 37,000 deaths in China in 2016 [3, 4]. For patients with locally advanced CC (LACC), chemoradiation followed by uterovaginal brachytherapy (BT) is the standard treatment [5–7]. Notably, for patients with refractory CC, such as those with large tumor volumes, adenocarcinoma, multiple para-aortic lymph node metastases, or curable stage IVB, conventional chemoradiotherapy with concurrent cisplatin had shown poor efficacy [8]. The Japan Gynecologic Oncology Group 1066 (JGOG1066) reported the results of a phase II trial that showed reductions in pelvic control rates for patients with stage III-IVA CC with diameters ≤50 mm and >70 mm from 85% to 54% after two years [9]. Insufficient radiation dose coverage of tumors with diameters greater than 50 mm was associated with a worse prognosis. Tumor recurrence and persistence were common in these patients [10–13]. A median overall survival (OS) of 9.3 months was reported after second-line systemic therapy, with an objective response rate (ORR) of 13.2% and a median progression-free survival (PFS) of 3.2 months [14].

According to the OUTBACK and GOTIC-002 lymphadenectomy followed by uracil and tegafur (LUFT) studies, adjuvant chemotherapy after radical chemoradiation did not improve PFS or OS and increased side effects [15, 16]. Recently, immune checkpoint inhibitors (ICIs) combined with concurrent chemoradiotherapy (CCRT) have become a research focus for high-risk LACC. The CALLA trial showed that durvalumab combined with CCRT failed to significantly improve PFS in high-risk LACC patients compared with CCRT alone [17]. The ENGOT-CX11/KEYNOTE-A18 study indicated that pembrolizumab combined with CCRT could improve PFS in LACC patients (hazard ratio [HR] ═ 0.70; *P* ═ 0.0020), but the current OS data are not yet mature, and the maintenance duration of treatment is prolonged [18]. Additionally, it is essential to fully determine the safety of immunotherapy and immunotherapy-based therapies and to focus on the impact of patients’ performance status, as measured by the Eastern Cooperative Oncology Group (ECOG), on the efficacy of immunotherapy and immunotherapy-based combination therapy [19–21].

**Figure 1.**
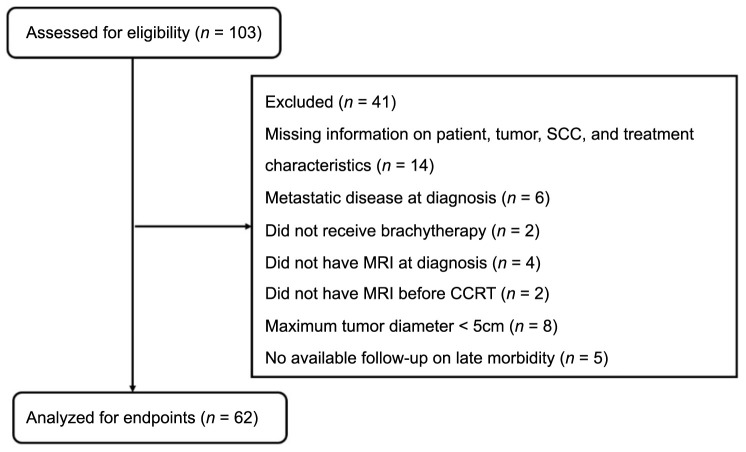
**Flow diagram displaying an overview of participants with detailed information regarding the excluded patients.** MRI: Magnetic resonance imaging; CCTR: Concurrent chemoradiotherapy; SCC: Squamous cell carcinoma antigen.

Previous studies have shown that higher vascular endothelial growth factor receptor 1/2 (VEGFR1/2) expression levels are associated with shorter PFS and OS [22]. The phase III clinical study GOG 240 showed that bevacizumab, a monoclonal antibody targeting VEGF, combined with chemotherapy significantly prolonged OS and PFS in patients with recurrent or metastatic CC [23]. The RTOG 0417 study reported that bevacizumab is safe and effective in LACC [24]. However, 82% of the patients included were in stages IB–IIB, and due to relatively small tumor volumes and superficial infiltration, these results had limited value for refractory CC treatment. To explore optimal treatment strategies for refractory CC, besides using the pegylated recombinant human granulocyte colony-stimulating factor (PEG-rhG-CSF) to prevent chemoradiotherapy-induced neutropenia [25], we also started clinical trials combining bevacizumab with neoadjuvant chemotherapy (NACT) and CCRT in 2016. It has been found safe and tolerable to combine bevacizumab with radical chemoradiotherapy, resulting in faster tumor regression and higher OS, local region-free survival rates (LRFS), distant metastasis-free survival (DMFS), and disease-free survival (DFS) rates [26]. Based on these promising results, our center further registered a multicenter, randomized controlled, prospective, phase II clinical study (NCT04138992) to evaluate the efficacy and safety of bevacizumab combined with CCRT in the radical treatment of LACC. The purpose of this study was to evaluate the long-term safety and efficacy of the combination of bevacizumab with NACT and CCRT in 62 patients with refractory CC.

## Materials and methods

### Patients

From January 2016 to December 2019, this study included patients with refractory CC treated by the Department of Radiation Therapy at the Xijing Hospital affiliated to the Air Force Military Medical University. Participants were meticulously selected based on strict inclusion and exclusion criteria.

The inclusion criteria were as follows: (1) a biopsy confirming primary CC; (2) patients aged 18–70 years; (3) a maximum tumor diameter of ≥ 5 cm; (4) an International Federation of Gynecology and Obstetrics (FIGO) stage IIB–IIIC2 and IVB stage with only supraclavicular and inguinal lymph node metastasis; (5) a pelvic MRI (3.0T) scanning performed pre-treatment, after NACR, and before BT; (6) no contraindications to bevacizumab; and (7) completion of the entire NACT + CCRT course.

The exclusion criteria were as follows: (1) individuals who have had cancer within the past five years; (2) those with inadequate clinical records and/or incomplete follow-up information; (3) patients with bladder or rectal invasion (Stage IVA); (4) a history of massive vaginal bleeding; (5) a history of hypertension and poor blood pressure control; and (6) patients with arteriovenous thrombosis or proteinuria.

Ultimately, 45 previously eligible patients and 17 patients from the prospective phase II study were enrolled (Figure 1). Clinical disease stages were classified according to the 2018 FIGO guidelines. The median age was 51 years (28–69 years). The median follow-up time was 49.8 months (12.3–82.7 months). The median pre-treatment tumor volume was 84.64 ± 53.15 cm^3^. In all patients, the tumor diameter was greater than 5 cm at diagnosis, with 25 patients (40.3%) having a tumor diameter greater than 6 cm. Additionally, 21 patients (33.9%) were at stage IIB–IIIB, eight patients (12.9%) at stage IIIC1, 19 patients (30.6%) at stage IIIC2, and 14 patients (22.6%) at stage IVB. Pelvic, para-aortic, supraclavicular, and inguinal lymph node metastases were observed in 41 patients (66.1%). Pelvic MRI (3.0T) scanning was performed before treatment, after NACR, and before BT. Patient characteristics are summarized in Table 1.

**Table 1:** Patient characteristics (*n* ═ 62)

**Parameters**	***n* (%)**	**Parameters**	***n* (%)**
*Age (years)*		*2018 FIGO stage*	
Median (range)	51 (28–69)	IIB-IIIB	23 (37.1)
*Tumor diameter (cm)*		IIIC1	6 (9.7)
≥ 6	25 (40.3)	IIIC2	19 (30.6)
5-6	37 (59.7)	IVB*	14 (22.6)
*Tumor volume (cm^3^)*		*Vaginal invasion*	
Median (range)	84.64 (45.6–264.8)	No	11 (17.8)
*No. of Ln*		Upper 1/3	18 (29.0)
≥ 3	30 (48.4)	Upper 1/2	18 (29.0)
1–2	11 (17.7)	Lower 1/3	15 (24.2)
0	21 (33.9)	*SCC (ng/mL)*	
*Ln necrosis*		Median (range)	8.7 (0.4–70)
Yes	12 (19.4)	*CEA (ng/mL)*	
No	50 (80.6)	Median (range)	3.6 (0.38–97.4)
*Ln short diameter (cm)*		*CA125 (U/mL)*	
<1	11 (17.7)	Median (range)	16.4 (1.71–382)
1–2	17 (27.4)	*Brachytherapy (Gy)*	Median (range)
>2	13 (20.9)	D90 for HR-CTV	82 (78.6–90)
*Ln volume (cm^3^)*		D2cc-rectum	70.8 (66.6–74.9)
Median (range)	13.5 (0–106.5)	D2cc-bladder	77.4 (70.9–84.5)
*Pathological type*		D2cc-sigmid	62.4 (56–73.8)
Squamous cell carcinoma	56 (90.3)	D2cc-intestine	60.8 (52.1–68.1)
Non-squamous cell carcinoma	6 (9.7)	*Cisplatin (days)*	
*Radiotherapy technology*		Median (range)	52 (39–79)
3DRT	12 (19.4)		
IMRT	50 (80.6)		

*Metastasis to supraclavicular and inguinal lymph nodes. Ln: Lymph nodes; FIGO: International Federation of Gynecology and Obstetrics; SCC: Squamous cell carcinoma antigen; CEA: Carcinoembryonic antigen; CA125: Cancer antigen 125; 3DRT: Three-dimensional conformal radiotherapy; IMRT: Intensity-modulated radiotherapy; D90: Dose delivered to 90% of the target volume; HR-CTV: High-risk clinical target volume; D2cc: Dose delivered to the most exposed 2 cubic centimeters.

### Chemotherapy and bevacizumab

Bevacizumab was prescribed at a dosage of 7.5 mg/kg in both the neoadjuvant and concurrent treatment regimens, administered every three weeks. The NACT protocol encompassed the use of docetaxel at 75 mg/m^2^ and cisplatin at 75 mg/m^2^, both given tri-weekly for a total of two cycles. Additionally, the CCRT protocol involved the delivery of cisplatin (DDP) at a dosage of 75 mg/m^2^, also on a tri-weekly schedule, for two cycles. These treatment plans were carefully designed to optimize the effectiveness and safety of bevacizumab in patients with refractory CC.

**Figure 2.**
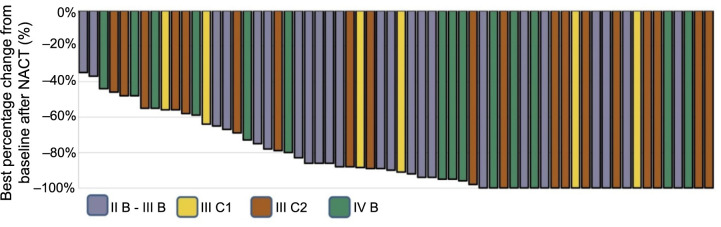
**Illustrating the best percentage change from baseline after NACT.** NACT: Neoadjuvant chemotherapy.

### Radiotherapy

A computed tomography (CT) simulation, utilizing a Brilliance CT Big Bore scanner, was conducted to obtain enhanced images of the pelvic and abdominal cavities. This process entailed acquiring slices with a thickness of 5 mm, capturing an area extending from the upper border of the kidneys to a point 5 mm beneath the ischial tuberosity. The external beam radiation therapy (EBRT) prescription consisted of administering 45–50 Gy in 25 fractions to the pelvis, and a dosage of 60–62.5 Gy in 25–29 fractions to metastatic lymph nodes, employing either volumetric modulated arc therapy or a three-dimensional conformal radiotherapy method. We used a Varian Clinac IX linear accelerator to emit a 6 MV X-ray beam and a Nucletron MicroSelectron-HDR Ir-192 remote BT instrument to deliver two fractions per week up to a total of 4–5 fractions. The dose for high-risk clinical target volume (HR-CTV) was set at ≥ 85 Gy (equivalent dose in 2 Gy fractions [EQD2]). The dose limits for organs at risk (OAR) were as follows: sigmoid colon 2cc ≤ 70–75 Gy, bladder 2cc ≤ 80–85 Gy, and rectum 2cc ≤ 65–75 Gy.

### Tumor measurement and response evaluation

MRI scans, specifically utilizing T2-weighted sequences, were employed to gauge tumor dimensions. The estimation of tumor volume adhered to the established formula: length × width × height × π/6, as documented in prior research [27]. To ascertain the percentage of tumor volume remaining post-intervention, the volume measured just before the initiation of EBRT or BT was divided by the original tumor volume. Tumor shrinkage efficiency was inferred through the calculation of 1 minus the residual tumor volume ratio. A clinical complete response (cCR) was declared when post-treatment T2-weighted MRI scans showed no discernible tumor presence, in accordance with the Response Evaluation Criteria in Solid Tumors (RECIST) version 1.1 guidelines.

### Ethical statement

Written informed consent was obtained from all patients for this study. The study was approved by the Ethics Review Committee of Xijing Hospital (protocol codes: KY20162017-2). All interventions conducted on human participants in this study adhered rigorously to the principles outlined in the 1964 Declaration of Helsinki, as well as its subsequent updates and amendments.

### Statistical analysis

Survival outcomes, including OS, LRFS, and DMFS rates, were meticulously estimated. For the statistical handling of these survival metrics and recurrence intervals, GraphPad Prism version 9.3.0 was utilized. Given that the normality assessment revealed that tumor volume and shrinkage rate data did not conform to a Gaussian distribution, descriptive statistics centered on the median values, supplemented by interquartile ranges, were used to accurately depict central trends and dispersion in the data.

## Results

### Patient information

A total of 62 patients with refractory CC were enrolled between January 2016 and December 2019. Of these, 45 patients were previously treated, and 17 patients were from the phase II prospective study (NCT04138992). The median follow-up time was 49.8 months (12.3–82.7 months). The median pre-treatment tumor volume was 84.64 ± 53.15 cm^3^. All patients had tumors larger than 5 cm in diameter at diagnosis, with 25 patients (40.3%) having tumors larger than 6 cm. Regarding the stage distribution, 21 patients (33.9%) were in stage IIB–IIIB, eight patients (12.9%) were in stage IIIC1, 19 patients (30.6%) were in stage IIIC2, and 14 patients (22.6%) were in stage IVB. Metastases in the pelvic, para-aortic, supraclavicular, and inguinal lymph nodes were discovered in 41 patients (66.1%).

### Tumor shrinkage

The median tumor volume of the whole group was 84.64 ± 53.15 cm^3^ before treatment. After NACT, the tumor volume reduced to 1.64 ± 13.15 cm^3^, resulting in a 97.55 ± 0.24% tumor shrinkage rate. Of all 62 patients, a cCR was observed in 22 patients (35.5%). The best percentage change from the baseline to after NACT is shown in Figure 2.

**Figure 3.**
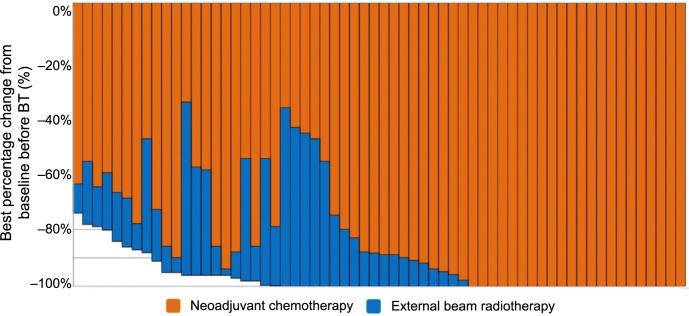
**Illustrating the best percentage change from baseline before BT.** BT: Brachytherapy.

The tumor volume further decreased to 0 ± 1.5 cm^3^ before BT, with the tumor shrinkage rate reaching 100.00 ± 0.05%. The cCR rate increased to 66.1% (41/62). The optimal reduction in tumor volume (>90%) before BT was observed in 88.7% of patients (55/62). The best percentage change from the baseline before BT is shown in Figure 3, where red represents NACT and blue represents EBRT. All patients received three-dimensional BT; one patient received interstitial BT, and 61 received intracavity BT. The HR-CTV dose exceeded 80 Gy in 58 patients.

### Survival

**Figure 4.**
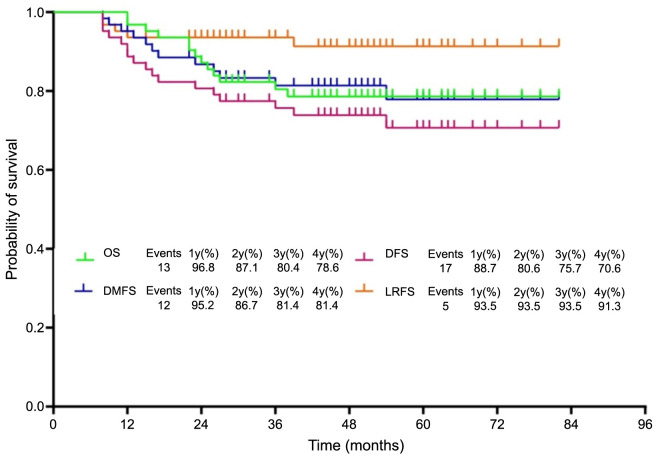
**Displaying the Kaplan–Meier curves for OS, DFS, DMFS, and LRFS.** OS: Overall survival; DFS: Disease-free survival; DMFS: Distant metastasis-free survival; LRFS: Local region-free survival.

The 1- to 4-year OS rates were 96.8%, 87.1%, 80.4%, and 78.6%. The LRFS rates were 93.5%, 93.5%, 93.5%, and 91.3%. The DMFS rates were 95.2%, 86.7%, 81.4%, and 81.4%. The DFS rates were 88.7%, 80.6%, 75.7%, and 70.6%, respectively (Figure 4).

### Treatment failure analysis

A total of 17 patients experienced treatment failure: five patients had local regional recurrence and 12 patients had metastasis. Among the patients with local recurrence, four had in-field cervical recurrences and one had out-of-field pelvic lymph node recurrence. Among the patients with distant metastasis, three had supraclavicular lymph node metastases, three had lung metastases, two had bone metastases, and four had multiple metastases. Of the 17 patients who failed treatment, 4 out of 5 with recurrence and 9 out of 12 with metastasis died.

### Side effects

Overall, the acute and late toxicities of bevacizumab and the entire treatment regimen were acceptable. The most common acute side effects were hypertension, nausea, diarrhea, and neutropenia. Twelve patients (19.4%) developed hypertension, five (8.1%) experienced nosebleeds, three (4.8%) had gum bleeding, two (3.2%) experienced hoarseness, and two (3.2%) had venous thrombosis. Grade 3/4 toxicities included 29 patients (46.8%) who developed acute hematotoxicities, and three patients (4.7%) who developed acute gastrointestinal toxicities. No grade 5 acute toxic reactions occurred in the entire group. Table 2 shows the acute and late adverse events.

**Table 2:** Acute and late adverse events

**Events**	**Grade 1-2**	**Grade 3-4**
	***n* (%)**	***n* (%)**
*Acute adverse events*		
Nausea	22 (35.5)	1 (1.6)
Diarrhea	38 (61.3)	2 (3.2)
Neutropenia	33 (53.2)	29 (46.8)
Venous thrombus	2 (3.2)	0
Hypertension	12 (19.4)	1 (1.6)
Nosebleeds	5 (8.1)	0
Bleeding gums	3 (4.8)	0
Hoarseness	2 (3.2)	0
*Late adverse event*		
Diarrhea	5 (8.1)	0
Hematochezia	6 (9.7)	0
Blood in urine	3 (4.8)	0
Vesicovaginal fistula	0	0
Rectovaginal fistula	0	0

## Discussion

The integration of bevacizumab with NACT and CCRT in our facility has yielded compelling clinical benefits for patients grappling with chemotherapy-resistant CC. Notably, this approach led to a cCR in 35.5% of patients following the completion of NACT, which escalated to 66.1% of cases prior to the initiation of BT. Encouragingly, the four-year survival outcomes included an OS rate of 78.6%, an LRFS rate of 91.3%, a DMFS rate of 81.4%, and a DFS rate of 70.6%. Aside from its efficacy, this therapeutic regimen has proven to be well-tolerated, with minimal safety concerns. Specifically, only a minority, constituting 4.8% of patients, encountered grade 3 gastrointestinal side effects, and reassuringly, no incidents of grade 5 adverse reactions were reported.

Although phase II studies GOG 240 and GOG 227C have documented bevacizumab’s survival benefits and safety profile in treating metastatic and recurrent cancer [23, 27], there is a scarcity of research specifically examining the combination of bevacizumab with CCRT in LACC, and even less so in patients with refractory CC. The present study showed that tumor volume was significantly reduced after NACT combined with bevacizumab. The mean tumor volume of the whole group was 84.64 ± 53.15 cm^3^ before treatment. After NACT, the mean tumor volume was 1.64 ± 13.15 cm^3^, and the tumor shrinkage rate was 97.55 ± 0.24%, with a cCR observed in 35.5% (22/62) of patients (Figure 2). Before BT, the mean tumor volume was 0 ± 1.5 cm^3^, and the tumor shrinkage rate was 100.00 ± 0.05%, with cCR observed in 66.1% (41/62) of patients (Figure 3). According to published data, a large tumor volume at diagnosis and a tumor volume greater than 30 cm^3^ indicated worse local control and generally required interstitial BT [28, 29]. In this study, due to the significant tumor shrinkage, the pre-BT tumor volume was reduced to 0 ± 1.5 cm^3^, resulting in interstitial BT being required for only one patient. The remaining patients all received intracavity BT. These results suggest that bevacizumab combined with NACT can increase the dose delivered to 90% of the target volume (D90) in patients with HR-CTV and simplify BT procedures by reducing the need for implantation. Furthermore, the EMBRACE2 study found that the optimal tumor volume reduction (>90%) before BT was independently associated with improved OS [30]. In the current study, the optimal tumor volume reduction (>90%) before BT was achieved in 88.7% of patients (55/62), which is significantly higher than the 59% reported in the EMBRACE2 study (Figure 3).

As for efficacy, four-year OS, LRFS, DMFS, and DFS rates from the current research were similar to those of RTOG 0417 [24]. However, while RTOG 0417 enrolled 82% of patients with stage IB–IIB disease, more than 60% of our patients were at higher stages, from IIIC2 to IVB. Tetsuya et al. [31] reported that in 18 patients with bulky (≥4 cm) and high-risk stage IIB–IVB LACC, the four-year OS and PFS were 87.8% and 81.6%, respectively. Similarly, interstitial BT was adopted in their study, which is time consuming, laborious, and difficult to perform. The EMBRACE1 study reported a 5-year local control rate of 92% in 1341 patients with MRI-based image-guided adaptive BT (IGABT) [32]. Other clinical data from large samples of CC patients receiving IGABT were similar to the results of this study [33–35].

Among the 17 patients who experienced treatment failure, five had local recurrences and 12 had metastases. Among those with recurrences, four had in-field recurrences, while one had a failure in the lymph nodes. Among the patients with distant metastases, three had supraclavicular lymph node metastasis, three had lung metastasis, two had bone metastasis, and four had multiple metastases. Of these 17 patients, four with recurrence and nine with metastasis died. Distant metastases, particularly in the supraclavicular lymph nodes and lungs, were the major cause of treatment failure, comprising 70.6% of the patients. This may be related to the excessive tumor load and advanced tumor stage in this group.

Cancer treatment has witnessed a revolutionary transformation with the introduction of immunotherapies that empower the immune system to target and eradicate cancerous cells. In CC, the overexpression of programmed death ligand 1 (PD-L1) is particularly rampant, affecting 55%–85% of squamous cell carcinomas and 64% of adenocarcinomas, underscoring its relevance as a therapeutic focal point [37]. Immunotherapy, specifically through ICIs, has shown promise in CC management, fueled by the recognized role of HPV in the disease. Advanced CC patients have been the subject of trials exploring vaccine-based interventions, adoptive T-cell transfers, and immunomodulatory strategies [38].

Notable outcomes include the KEYNOTE-158 trial, where PD-L1-positive (combined positive score [CPS] ≥ 1) CC patients achieved an ORR of 14.6% and a disease control rate (DCR) of 32.9%, contrasting with no responses in PD-L1-negative cohorts [39]. In the CHECKMATE-358 study, involving 19 CC patients, nivolumab administration resulted in a 26.3% ORR and a 68.4% DCR among PD-L1-positive subjects [40]. The EMPOWER-Cervical 1 trial, enrolling 608 advanced CC patients, demonstrated that cemiplimab, administered as second or third-line therapy, improved survival, with a median OS of 12 months compared to 8.5 months for those receiving standard chemotherapy, with efficacy closely tied to PD-L1 expression [41]. Encouraged by these successes in later-stage treatments, researchers began exploring ICIs in primary therapeutic contexts. The KEYNOTE-826 trial paired pembrolizumab with platinum-based chemotherapy, with or without bevacizumab, for previously untreated advanced CC patients, noting superior PFS and OS, especially among PD-L1-positive patients, with a 2-year OS rate of 53% vs 41.7% in the control group [42]. Reflecting these advancements, the National Comprehensive Cancer Network (NCCN) now recommends pembrolizumab in combination with first-line platinum-based chemotherapy, with or without bevacizumab, for recurrent or metastatic PD-L1-positive CC. Anticipation also surrounds the long-term outcomes of the ENGOT-CX11/KEYNOTE-A18 trial, which is examining the integration of ICIs with chemoradiotherapy for LACC patients with lymph node involvement, further solidifying the evolving landscape of CC immunotherapy.

In summary, LACC is prone to residual disease, recurrence, or metastasis after standard CCRT. Patients with high-risk factors, such as pelvic wall invasion, are particularly challenging, making standard treatment no longer suitable for these high-risk individuals. Improving clinical efficacy for these patients has become a significant challenge. In this study, individualized treatment with bevacizumab combined with NACT and CCRT was administered. Retrospective clinical studies showed that bevacizumab combined treatment had better therapeutic effects and tolerable side effects. Larger, higher level clinical studies are currently underway.

Efficiently screening high-risk LACC is a critical gap in this study. Our research indicates that spatial and multitask attention networks can better predict the efficacy of radiotherapy and chemotherapy for CC [43]. To further improve prediction accuracy and clarify the relationship between tumor heterogeneity and radiotherapy efficacy, we designed a heterogeneity characterization network. This network characterizes tumor heterogeneity in habitat imaging and effectively screens high-risk LACC patients who are at risk of tumor residual, recurrence, and metastasis after standard treatment.

This study has several limitations. Firstly, we observed only a small number of participants. Secondly, 45 of the enrolled patients were retrospectively analyzed, although they were strictly screened. Simultaneously, our center registered a multi-center, randomized controlled, prospective phase II clinical study (NCT04138992), which is investigating the efficacy and safety of bevacizumab combined with CCRT in the radical treatment of LACC. The multicenter clinical trial has completed patient recruitment and is ongoing. More results will be reported in the future.

## Conclusion

Bevacizumab combined with NACT and CCRT improved tumor regression and cCR rates and reduced the usage of interstitial BT in refractory CC. Four-year OS and PFS were higher than previously reported, while the incidence of complications did not increase during long-term follow-up. This new treatment strategy has been demonstrated to be practical and promising for addressing clinical challenges.
